# Light-mediated activation of *PpPSY* enhances β-carotene accumulation in pear fruit peel

**DOI:** 10.3389/fpls.2025.1542830

**Published:** 2025-02-28

**Authors:** Li Zhang, Wei Du, Junfan Tu, Hongyan Zhu, Xianming Li

**Affiliations:** Hubei Key Laboratory of Germplasm Innovation and Utilization of Fruit Trees, Institute of Fruit and Tea, Hubei Academy of Agricultural Sciences, Wuhan, China

**Keywords:** light, pear, carotenoid, PSY, AGL8, LFY

## Abstract

Light is a key environmental factor that regulates fruit development and influences several important quality traits, including pericarp color. In pear fruits, carotenoids are the primary determinant of pericarp color. However, the molecular mechanisms underlying light-mediated carotenoid accumulation remain poorly understood. This study investigated the carotenoid contents in the peels of non-bagged (light-exposed) and bagged (shaded) pear fruits (Cuiguan, *Pyrus pyrifolia*) and revealed a significant differences in β-carotene content between the two treatments. Transcriptome analysis revealed that the expression of *phytoene synthase* (*PSY*) was downregulated in bagged fruits, highlighting the regulatory role of *PSY* in carotenoid metabolism. To further validate this, we transiently overexpressed *PSY*, which resulted in a marked increase in β-carotene levels at the injection site. Conversely, transient silencing of *PSY* led to a significant reduction in the β-carotene content, confirming the pivotal role of *PSY* in regulating β-carotene accumulation. Promoter analysis revealed that agamous-like 8 (AGL8) directly binds to the *PSY* promoter to activate its transcription. Protein−protein interaction assays demonstrated that AGL8 interacts with LEAFY (LFY), thereby increasing *PSY* expression. In conclusion, the AGL8-LFY complex coactivates *PSY* expression, regulating β-carotene accumulation in pear fruit. This study provides new insights into the regulatory network governing fruit peel coloration, with potential applications for cultivation strategies to improve fruit quality.

## Introduction

Carotenoids accumulate abundantly in the pericarp, contributing to fruit pigmentation, as observed in tomato (Fraser et al., 1999), citrus (Peng et al., 2013), and melon (Qin et al., 2011). In addition to their role in coloration, carotenoids perform multiple physiological functions such as stress resistance (Edge et al., 1997), act as precursors for plant hormones (Schwartz et al., 1997; Alder et al., 2012), attract pollinators (Bartley and Scolnik, 1995), and contribute to photosynthesis (Frank and Cogdell, 1996; Niyogi et al., 1997; Holt et al., 2005).

Carotenoid biosynthesis represents a tightly regulated metabolic pathway in which phytoene synthase (PSY) governs the primary rate-limiting step, serving as the central regulatory node for this essential biochemical process. PSY converts geranylgeranyl diphosphate (GGPP) into phytoene, initiating the carotenoid biosynthetic pathway (Summers et al., 1993; Kachanovsky et al., 2012). In orange-fleshed melon, the accumulation of carotenoids during ripening is correlated with the upregulation of key genes, such as *PSY*, *lycopene beta-cyclase* (*LCYb*), and *phytoene desaturase* (*PDS*) (Chayut et al., 2015). Similar gene activation patterns have been reported in tomato and watermelon, where ripening triggers the expression of *PSY* and *PDS*, resulting in increased carotenoid levels (Giuliano et al., 1993; Fraser et al., 1994).

The regulation of carotenoid biosynthesis by light is a complex physiological process. Light-responsive transcription factors interact with promoter elements to regulate the expression of genes involved in carotenoid biosynthesis, ultimately influencing carotenoid accumulation. In *Arabidopsis thaliana*, light signaling represses carotenoid biosynthesis through phytochrome interacting factor (PIF) proteins that directly suppress *PSY* expression (Toledo-Ortiz et al., 2014). In tomato, mutants with reduced expression of the transcription factor *elongated hypocotyl 5 (HY5)* exhibit a weakened response to light signals, leading to lower *PSY* expression and impaired carotenoid biosynthesis (Liu et al., 2004). In citrus, MADS3 directly binds to the promoters of *PSY* and *LCYb*, positively regulating their transcription and increasing the carotenoid content in the fruit peel (Zhu et al., 2023). Agamous-like 8 (AGL8), a member of the MADS-box gene family, is part of the AGAMOUS-like subfamily, which plays significant roles in flower and fruit development (Benfey and Chua, 1990; Ferrándiz et al., 2000). However, it remains unclear whether *AGL8* directly or indirectly responds to light signals and regulates downstream carotenoid biosynthesis genes.

Pear fruit peels present a range of colors, including red, yellow, green, and white. While many existing studies have focused on anthocyanins as determinants of red coloration, the role of carotenoids in peel coloration has been largely overlooked (Bai et al., 2019; Ni et al., 2023; Gao et al., 2024). This study addresses the gap in understanding the molecular mechanisms of light regulation of peel coloration in pear. Significant differences in carotenoid contents were detected between bagged and non-bagged pear fruits. Transcriptomic sequencing identified *PSY* as a differentially expressed gene, and HPLC analysis revealed that the β-carotene content significantly differed between bagged and non-bagged fruit peels. Transient overexpression and silencing of *PSY* confirmed its regulatory role in carotenoid biosynthesis. Interaction analysis demonstrated that AGL8 interacts with leafy (LFY) to coactivate *PSY* expression. Our results provide insights into the molecular mechanisms underlying carotenoid accumulation in pear fruit, offering potential applications for molecular breeding and fruit quality improvement.

## Materials and methods

### Plant materials

For this study, nine-year-old healthy and actively growing pear plants (*Pyrus pyrifolia* ‘Cuiguan’) were selected from the Hubei Academy of Agricultural Sciences, Wuhan, P. R. China. On May 1^st^, fruits were bagged with paper bags that were yellow on the outside and black on the inside. On July 10th, thirty fruits from each treatment were randomly harvested for analysis. For transient transformation experiments, *Nicotiana benthamiana* plants were grown in a climate-controlled chamber at 22°C with a 16-hour light/8-hour dark photoperiod. Leaves from seven-week-old *N. benthamiana* plants were used for sample injections.

### RNA extraction, cDNA library construction, and RNA-seq

Total RNA was extracted from the fruit peel using the EASYspin Plant Extraction Kit (RN40, Aidlab Biotechnologies Co., Ltd., China.), following the manufacturer’s instructions. RNA purity and concentration were assessed using a NanoDrop 2000 spectrophotometer, and RNA integrity was evaluated with an Agilent 2100 or LabChip GX system. The RNA samples were submitted to Biomarker Technologies (Beijing, China) for paired-end RNA sequencing. cDNA libraries were prepared using the NEBNext^®^ UltraTM RNA Library Prep Kit (NEB, USA) and sequenced on the Illumina NovaSeq 6000 platform. To ensure high-quality data, the raw reads were filtered to eliminate adapter sequences and low-quality reads. Cleaned reads were aligned to the P. pyrifolia v1.0 reference genome (Gao et al., 2021) using HISAT2. Gene expression levels were quantified on the basis of fragments per kilobase of transcript per million fragments mapped (FPKM) values.

Differentially expressed genes (DEGs) were identified using DESeq2, with a threshold of |log2 fold change| ≥ 1.5 and a false discovery rate (FDR) < 0.05. The DEGs were subjected to Gene Ontology (GO) and Kyoto Encyclopedia of Genes and Genomes (KEGG) enrichment analyses, with significant pathways identified at a q- value < 0.05. Visualizations, including heatmaps, bubble charts, and principal component analysis (PCA), were generated using R.

### Gene expression and immunoblot analysis

Total RNA was extracted using the FastPure Universal Plant Isolation Kit (RC411, Vazyme Biotech Co., Ltd., China.), following the manufacturer’s protocol. The RNA quality was assessed with a Denovix 2017 spectrophotometer (Bio-SUN). Reverse transcription was performed using the HiScript IIQ RT SuperMix for qPCR (+gDNA wiper) kit, with ACTIN (MSTRG.11298.4) used as the normalization housekeeping gene. Gene expression was analyzed via reverse transcription-quantitative PCR (RT-qPCR) on a QuantStudio™ 6 Flex real-time PCR system (Applied Biosystems, USA) in 384-well plates. The data were analyzed using the 2-ΔΔCt method (Bustin et al., 2009). All primers used are listed in **Supplementary Table S1**.

Protein was extracted using a protein extraction kit (Solarbio, Beijing, China), and 30 µg of protein per sample was loaded onto gels for electrophoresis. Proteins were transferred to polyvinylidene fluoride (PVDF) membranes (0.45 µm, Millipore). Immunoblotting was conducted using a GFP antibody (ABclonal: AE012, Wuhan, China), followed by incubation with a secondary anti-mouse IgG (H+L) antibody (ABclonal: AS014, Wuhan, China). The detection of the actin protein was performed using an anti-β-actin mouse monoclonal antibody (ABclonal: AC009; Wuhan, China).

### Gene vector construction and transformation

For transient overexpression in ‘Cuiguan’ pear fruits, the full-length coding sequence (CDS) of *PSY* was amplified, cloned and inserted into the PRI101-GFP vector, following the infection protocol described by Gu et al. (2024). For the VIGS-mediated gene silencing vector, a 200-300 bp fragment of the *PSY* CDS was amplified, digested with EcoRI and SmaI, and inserted into the TRV2 vector using 2 × Ezmax Universal CloneMix (Tolobio, 24305). *Agrobacterium tumefaciens* strain GV3101 carrying TRV1 and TRV2 constructs was infiltrated into pear fruits at a 1:1 ratio, as outlined by Cao et al. (2024). All primers used are listed in **Supplementary Table S1**.

### Transcription activation analysis

The full-length CDS of *AGL8* was subsequently cloned and inserted into the pGBKT7-BD vector using EcoRI and BamHI. The empty and fusion vectors were transformed into the yeast strain AH109, following the protocol described by Zhang et al. (2023). All primers used are listed in **Supplementary Table S1**.

### Quantitation of chlorophyll and carotenoid content

Fresh samples (0.5 g) of fruit peels and flesh were powdered. These samples were subsequently placed in 10 mL centrifuge tubes containing ethanol and acetone (v/v= 2/1). After 12 hours, the supernatant was transferred to a 96-well plate. The absorbance was measured at A_663_ for chlorophyll a, A_645_ for chlorophyll b, and A_470_ for carotenoids. Calculations were performed using the method previously described by Zhang et al. (2023).

### Carotenoid extraction and HPLC analysis

Fruit peel samples were lyophilized using a lyophilizer (catalog no. 7960070; LABCONCO FreeZone, USA). A total of 1 g of dried sample was analyzed using high-performance liquid chromatography (HPLC) (e2695; Waters, USA), following the method described by Zheng et al. (2019).

### Dual-luciferase and split-LUC assays

The CDS of *AGL8* was cloned and inserted into the pGreenII-62-SK-LUC vector (effector), and promoter fragments were inserted into the pGreenII-0800-LUC vector (reporter) using SalI and KpnI or KpnI and NcoI, respectively. *Agrobacterium* strains carrying both constructs were mixed at a 10:1 ratio and infiltrated into *N. benthamiana* leaves, as described by An et al. (2024). The pGreenII-62-SK-LUC vector without the *AGL8* gene was used as a negative control. Three days post-infiltration, firefly and Renilla luciferase activities were measured using dual luciferase assay reagents (Promega) on an Infinite M200 plate reader (Tecan). Moreover, split-LUC was also performed, as described by Zhang et al. (2022). The LUC/REN ratio was used to calculate transactivation activity. All primers used are listed in **Supplementary Table S1**.

### Fluorescence complementary imaging

The CDSs of *AGL8* and *LFY* were subsequently cloned and inserted into the JW771 and JW772 vectors, respectively. These vector pairs were subsequently co-transformed into *N. benthamiana* leaves. Three days after transformation, LUC fluorescence was detected using dual luciferase assay reagents (Promega) with a Vivo Plant Imaging System (NightShade LB 985, Berthold, Bad Wildbad, Germany). All primers used are listed in **Supplementary Table S1**.

### Statistical analysis

Statistical analysis was performed on data from three biological replicates. The values are expressed as the means ± SEs. The data were analyzed using Origin (version 2018), Excel (version 2010), R (version 4.1) and SPSS (version 26.0). Student’s *t* test was used to compare pairs of groups, and statistical significance was determined with thresholds of *P* < 0.05 and *P* < 0.01.

## Results

### Phenotype and carotenoid content differences between non-bagged and bagged fruits

**Figure 1:**
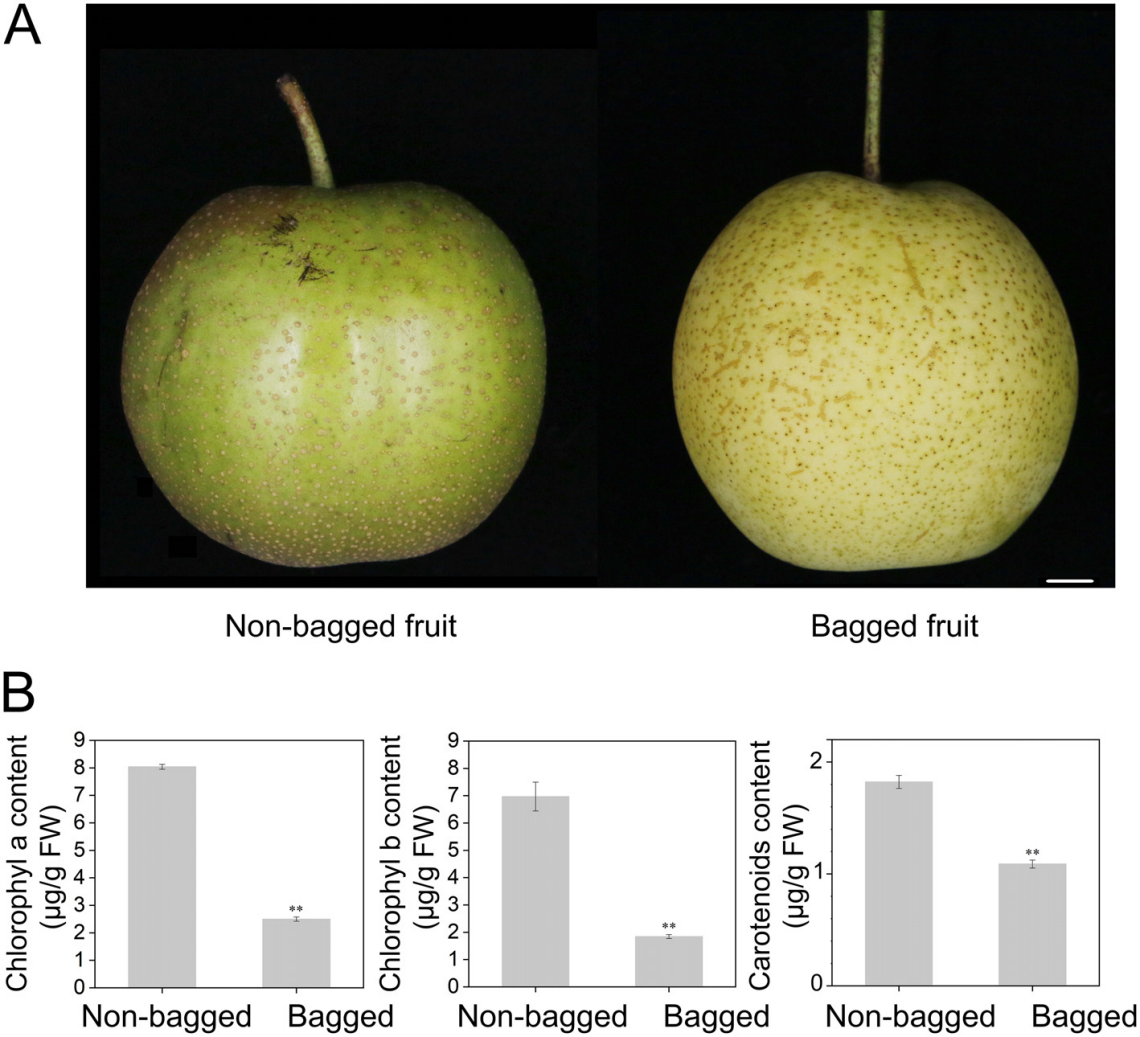
Color phenotypes and carotenoid and chlorophyll contents of bagged fruits and non-bagged fruits. **(A)** Photographs of representative pear fruit colors. Bar, 1 cm. **(B)** Measurement of chlorophyll and carotenoid concentrations in bagged fruits and non-bagged fruits. Asterisks indicate statistical significance in **(B)** as determined by Student’s *t* test: **P < 0.01. FW, fresh weight.

Compared with non-bagged fruits, bagged fruits display distinct peel colors (**Figure 1A**). Chlorophyll (a and b) and carotenoid contents were measured in both the peel and flesh. The results indicated that the chlorophyll and carotenoid contents were significantly greater in non-bagged fruits than in bagged fruits (**Figure 1B**). However, no chlorophyll or carotenoid content was detected in the fruit flesh.

### Transcriptomic and metabolic differences in non-bagged and bagged pear fruit peels

RNA-seq was performed on samples from both non-bagged and bagged fruits. The total number of clean reads ranged from 20,981,433 to 22,664,610, with an average Q30 value of 90.45% and a GC content ranging from 45.91% to 46.30% (**Supplementary Data Set S1**). The FPKM density distribution comparison chart for each sample revealed that most gene expression levels were concentrated between 0.1 and 10 (**Supplementary Figure S1A**). The PCA results (PC1 explained 72.0%, and PC2 explained 8.5%) demonstrated good repeatability within each group (**Supplementary Figure S1B**). A correlation heatmap confirmed strong consistency among three biological replicates of each treatment group (**Supplementary Figure S1C**).

**Figure 2:**
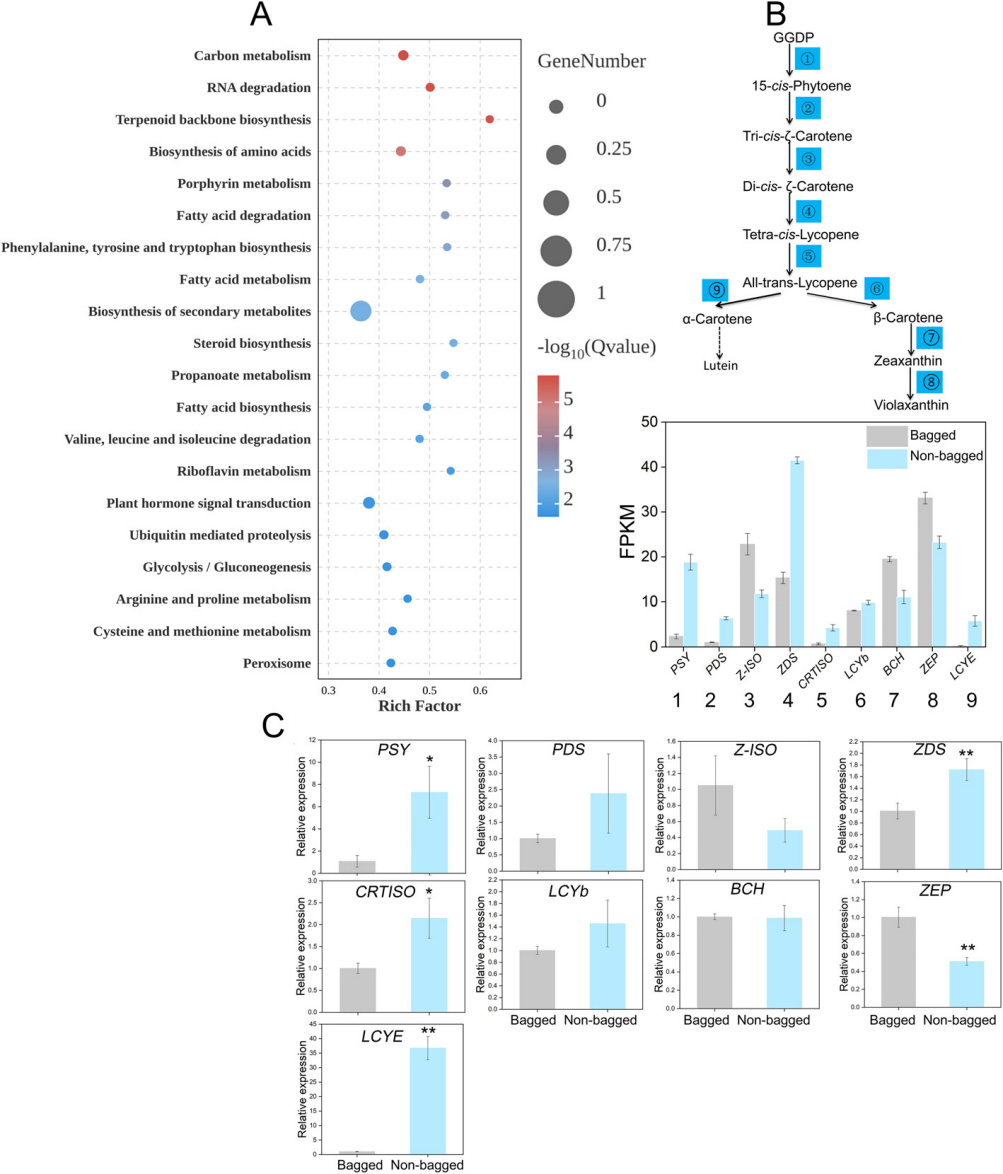
Differential gene analysis between bagged and non-bagged pear fruit peels. **(A)** KEGG enrichment analysis between bagged and non-bagged pear fruit peels. The circle size indicates the DEG count, and the circle color indicates the q value. **(B)** Expression levels of genes related to the *de novo* synthesis of carotenoids. **(C)** Relative expression of nine genes in the bagged and non-bagged pear fruit pericarp. Asterisks indicate statistical significance in **(C)** as determined by Student’s *t* test: **P < 0.01, *P < 0.05.

A total of 14,643 DEGs were identified (log2fold change≥ 1.5; P-value≤ 0.05; **Supplementary Data Table S2**). A heatmap of the DEGs revealed distinct clusters of upregulated and downregulated genes in the non-bagged and bagged samples (**Supplementary Figure S1D**). GO enrichment analyses highlighted significant enrichment of DEGs in processes, such as ‘single-organism process’ (GO:0044699) and ‘single-organism cellular process’ (GO:0044763). Bagging was classified as a non-biological stress process, and the GO enrichment analysis revealed that the DEGs were also enriched in the ‘response to abiotic stimulus’ process (**Supplementary Figure S2**). KEGG analysis revealed enrichment in pathways including ‘Biosynthesis of amino acids’, ‘Biosynthesis of secondary metabolites’, and ‘Plant hormone signal transduction’ (**Figure 2A**).

Nine key genes, including *PSY*, *PDS*, *15-*cis*-zeta-carotene isomerase* (*Z-ISO*), *zeta-carotene desaturase* (*ZDS*), *carotenoid isomerase* (*CRTISO*), *LCYb*, *β-carotene hydroxylase* (*BCH*) and *zeaxanthin epoxidase* (*ZEP*), were identified from the DEG data. Except for *Z-ISO*, *BCH*, and *ZEP*, the FPKM values of the remaining six genes were greater in the non-bagged fruits (**Figure 2B**). RT-qPCR was used to validate the expression levels of these nine genes, and the results were highly consistent with the FPKM trends (**Figure 2C**).

**Figure 3:**
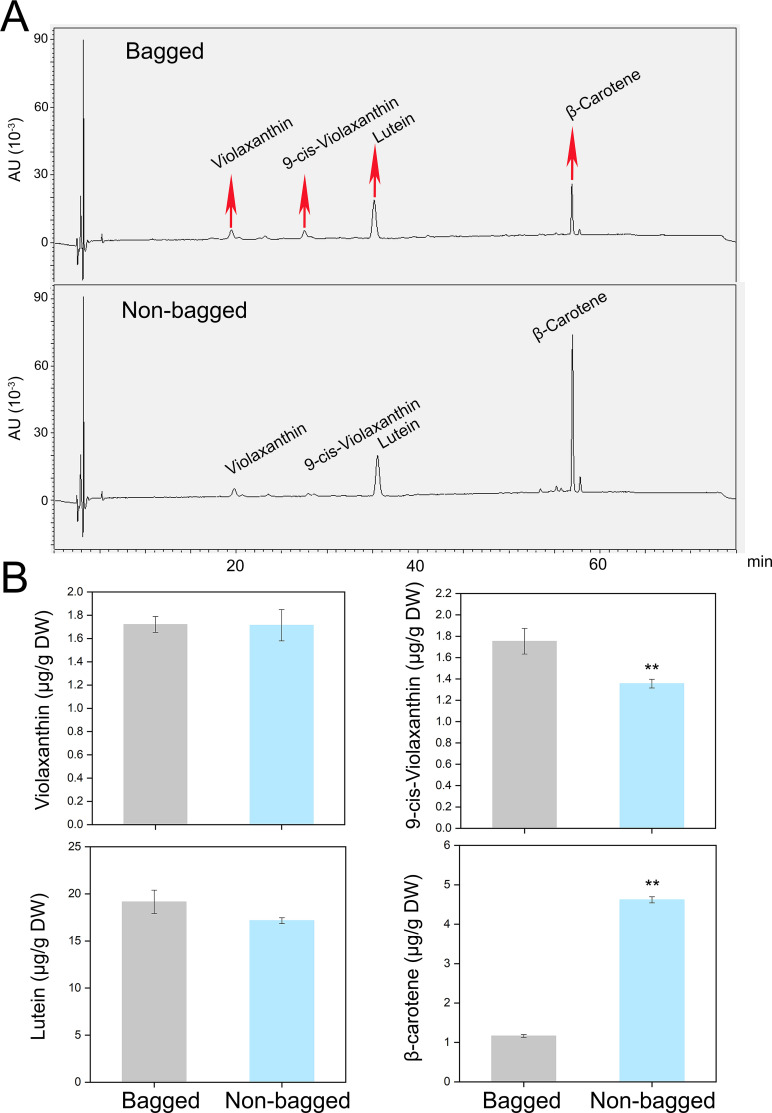
HPLC analysis of bagged and non-bagged pear fruit peels. **(A)** HPLC profile of carotenoids from peels. The peaks indicated with red arrows at 20 min, 28 min, 36 min, and 57 min represent violaxanthin, 9-cis- violaxanthin, lutein, and β-carotene, respectively. **(B)** Contents of the four metabolites in bagged and non-bagged pear fruit peels. Asterisks indicate statistical significance in **(B)** as determined by Student’s *t* test: **P < 0.01. DW, dry weight.

To correlate the transcriptional data with the metabolic changes, the carotenoid metabolite levels were quantified using HPLC. Only four carotenoid metabolites, violaxanthin, 9-cis-violaxanthin, lutein, and β-carotene, were detected in the peel. While the content of violaxanthin was similar between non-bagged and bagged fruits, the contents of 9-cis-violaxanthin and lutein were significantly greater in bagged fruits. In contrast, the β-carotene content was significantly greater in non-bagged fruits (**Figures 3A, B**).

### Transient overexpression and silencing of the *PSY* gene alter the carotenoid content

**Figure 4:**
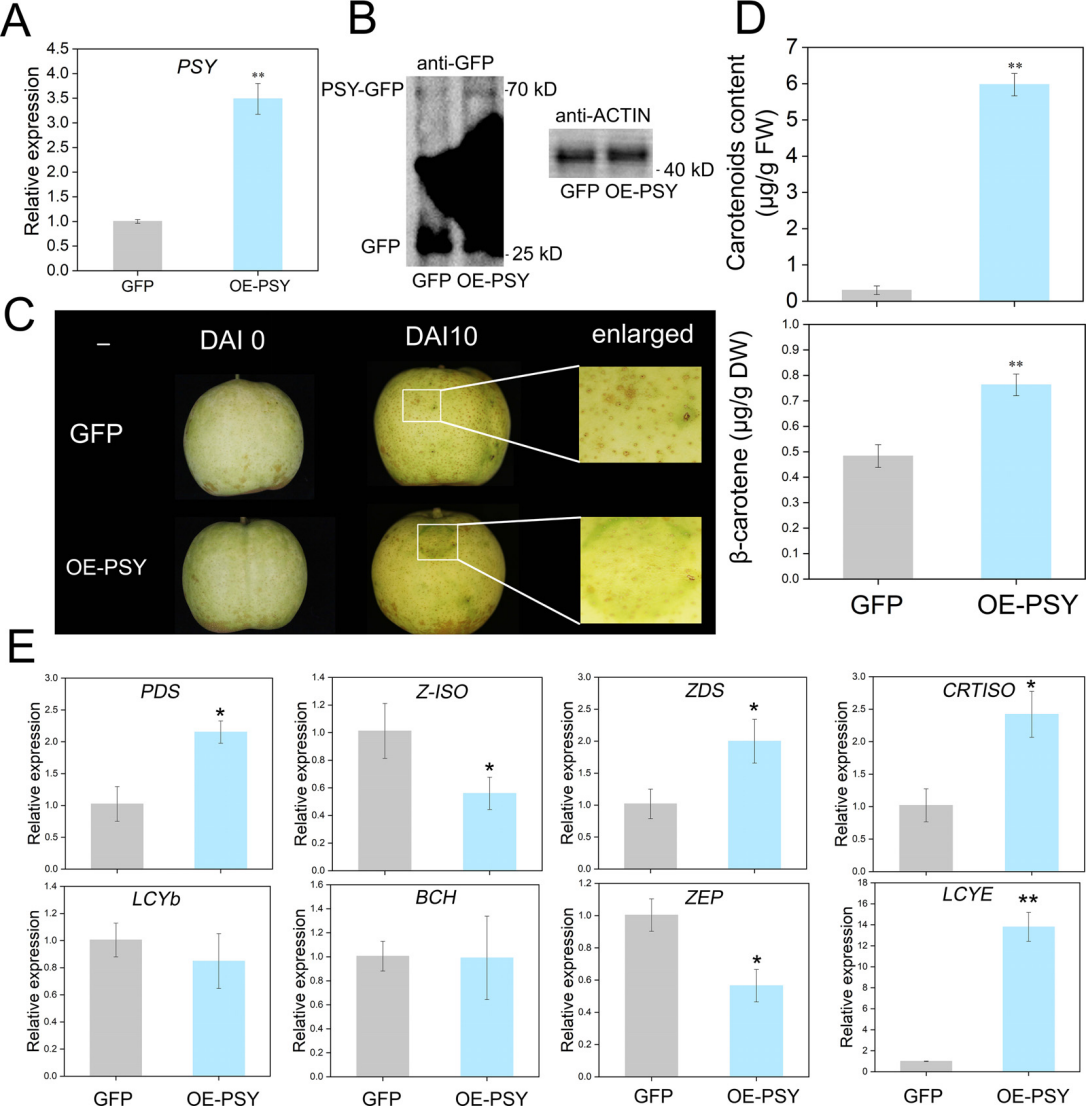
Phenotypic and gene expression changes caused by transient overexpression of *PSY* in pear fruits. **(A)** Relative expression of *PSY* in the GFP control and overexpression fruits. **(B)** Western blot (WB) analysis of control and GFP-overexpressing fruits. GFP (23.8 kDa) and GFP-PSY proteins (70.77 kDa). **(C)** Transient overexpression of *PSY* promotes carotenoid synthesis in pear fruits. **(D)** Measurement of carotenoid contents in the GFP control and overexpression fruits. **(E)** Relative expression of eight genes in the GFP and PSYOE fruits. Asterisks indicate statistical significance in **(A, D, E)** as determined by Student’s *t* test: **P < 0.01, *P < 0.05.

The PRI101-PSY-GFP vector was constructed to verify the function of *PSY*. Prior to injection, the carotenoid content in the pear peel did not significant differ (**Supplementary Figure S3**). Ten days after injection, transcriptional analysis and Western blotting confirmed successful *PSY* overexpression (**Figures 4A, B**). Compared with control fruits, positive fruits presented yellow–green coloration (**Figure 4C**). Analysis of the injection areas revealed higher β-carotene and other carotenoid levels in positive fruits than in control fruits (**Figure 4D**; **Supplementary Figure S4**). To determine whether the upregulation of *PSY* expression positively regulates the transcription levels of downstream genes, qRT-PCR was used to validate the expression levels of the remaining eight genes. The experimental results indicated that the expression trends of the eight genes were highly consistent with the transcriptome data (**Figure 4E**).

**Figure 5:**
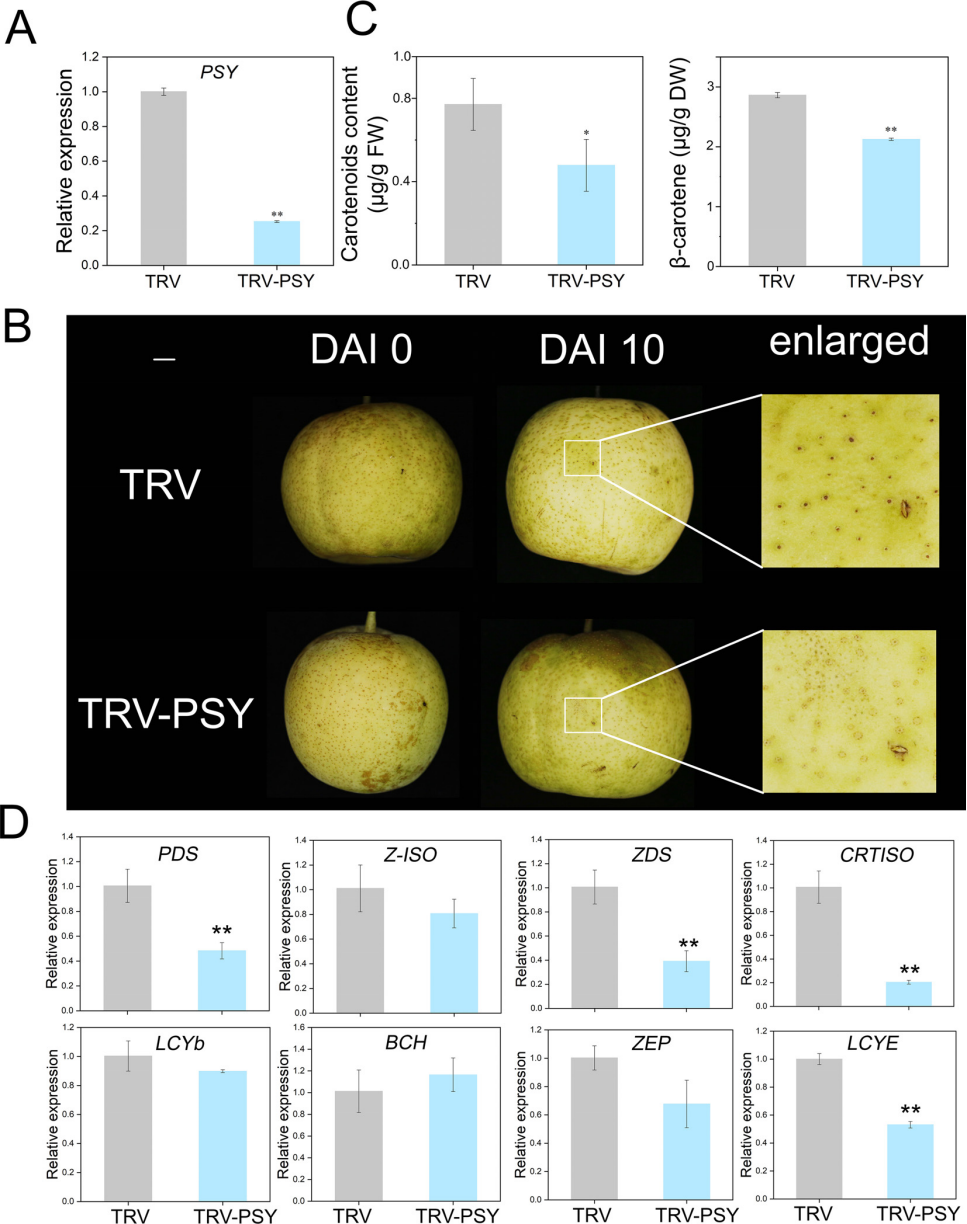
Phenotypic and gene expression changes caused by transient silencing of *PSY* in pear fruits. **(A)** Relative expression of *PSY* in the TRV control and TRV-PSY fruits. **(B)** Transient silencing of *PSY* inhibits carotenoid synthesis in pear fruit. **(C)** Measurement of carotenoid concentrations in the TRV control and TRV-PSY fruits. **(D)** Relative expression of seven genes in the TRV and TRV-PSY fruits. Asterisks indicate statistical significance in **(A, C, D)** as determined by Student’s *t* test: **P < 0.01 and *P < 0.05.

The TRV-PSY vector was constructed to confirm the function of *PSY*. Carotenoid levels in fruits were measured before injection, and no significant differences were detected (**Supplementary Figure S5**). Ten days after injection, successful silencing of *PSY* was confirmed (**Figure 5A**). Compared with the control fruits, the positive fruits presented a lighter color (**Figure 5B**). The contents of β-carotene and other carotenoids were significantly lower in positive fruits than in control fruits (**Figure 5C**; **Supplementary Figure S6**). RT-qPCR validated the expression of the remaining eight genes, which aligned with the transcriptomic data trends (**Figure 5D**).

### AGL8 and LFY proteins interact to coregulate the transcription of *PSY*

**Figure 6:**
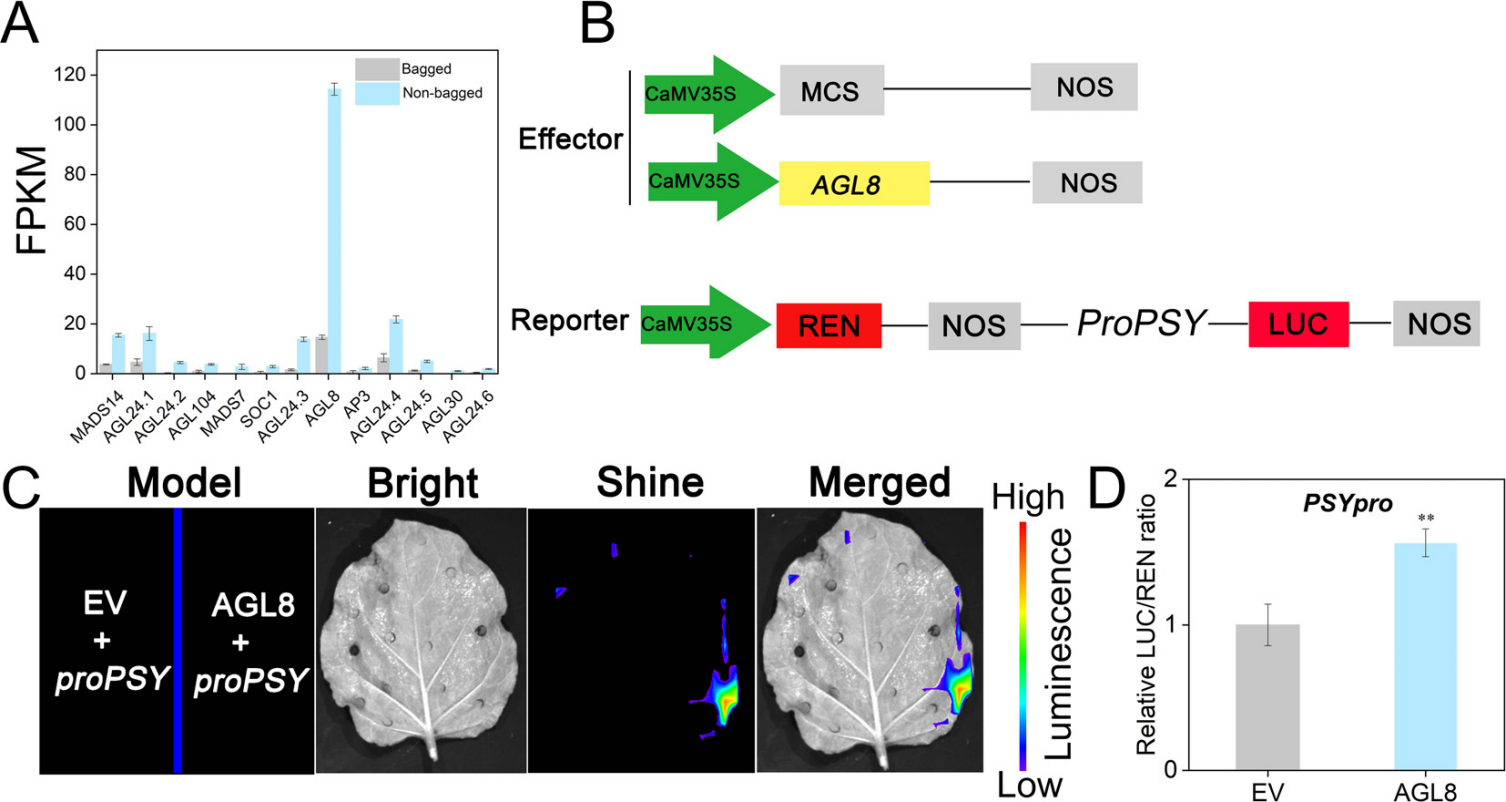
AGL8 positively regulated *PSY* to modulate transcript levels. **(A)** FPKM values of candidate genes. **(B)** Diagram of vector construction. MCS, multiple cloning site; LUC, firefly luciferase activity; REN, Renilla luciferase; NOS, nopaline synthase. **(C)** The interaction between AGL8 and *PSY* in the split-luciferase assays is shown. **(D)** The Dual Luciferase Assay System was utilized for the detection of AGL8 targeting *PSY*. EV, empty vector. Asterisks indicate statistical significance in **(C)** as determined by Student’s *t* test: **P < 0.01.

Transcriptomic analysis revealed 12 upregulated MADS-box genes (**Figure 6A**). Among these genes, *AGL8* presented the highest FPKM value and was consistently upregulated with *PSY*, suggesting its key role in carotenoid biosynthesis. The AGL8 protein was fused to an effector vector, and the *PSY* promoter (1988 bp) was linked to a reporter vector (**Figure 6B**). Fluorescence imaging revealed that AGL8 directly binds to the *PSY* promoter and activates its transcription (**Figure 6C**). This activation was further confirmed by a dual-luciferase assay (**Figure 6D**).

To test the transcriptional activation activity of *AGL8*, its CDS was cloned and inserted into the pGBKT7-BD vector and transformed into the yeast strain AH109. The positive strains grew similarly to the controls in single, double, and quadruple dropout media (**Supplementary Figure S7**), indicating that *AGL8* lacks intrinsic transcriptional activation activity and may require interaction partners for coactivation.

**Figure 7:**
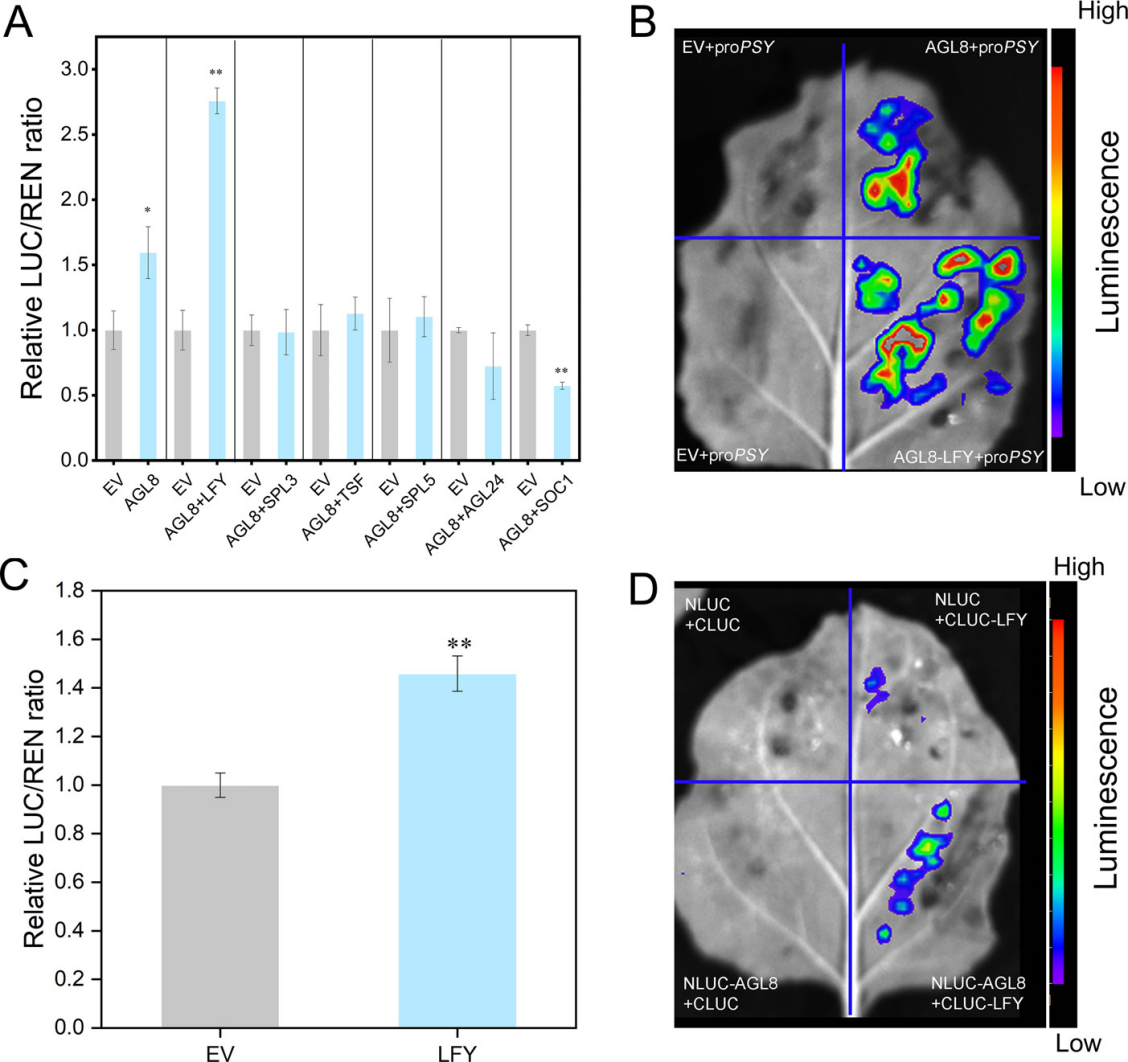
AGL8 interacts with the protein LFY to coactivate the transcription of *PSY*. **(A)** A dual-luciferase assay system was used to detect the targeting of *PSY*. Agamous-like (AGL), leafy (LFY), squamosa promoter binding protein-like (SPL), twin sister of ft (TSF), suppressor of overexpression of co (SOC). **(B)** A split-LUC assay was performed to identify the coactivation of the *PSY* promoter. **(C)** A dual-luciferase assay system was used for the targeting of *PSY* by LFY. **(D)** Interaction between AGL8 and LFY. Asterisks indicate statistical significance in **(A, C)** as determined by Student’s *t* test: **P < 0.01 and *P < 0.05.

Protein interaction analysis predicted *LFY* as a coactivator of *AGL8* (**Supplementary Figure S8**). Dual-luciferase assays revealed that, compared with *AGL8* alone, the coexpression of *AGL8* and *LFY* significantly increased *PSY* promoter activation (~2.76-fold) (**Figure 7A**). Split-LUC assays confirmed the interaction between *AGL8* and *LFY* in coactivating the *PSY* promoter (**Figure 7B**). Further dual-luciferase experiments revealed that *LFY* alone weakly activated the *PSY* promoter, whereas coinjection with *AGL8* strongly increased activation. LCI confirmed the interaction between *AGL8* and *LFY* (**Figure 7D**).

## Discussion

### Light promotes carotenoid accumulation in pear fruit peels

Light plays a pivotal role in fruit development and metabolic processes (Pizarro and Stange, 2009). In citrus fruits, LED red light treatments have been shown to increase the β-cryptoxanthin content in the peel (Ma et al., 2012). Conversely, bagging treatments, which limit light exposure, have been shown to affect carotenoid accumulation (Lopez et al., 1986; Lado et al., 2019) and anthocyanin biosynthesis (Zhou et al., 2010).

In this study, ‘Cuiguan’ pear fruits were subjected to bagging treatment for 70 days. The carotenoid content was significantly greater in the peels of non-bagged fruits than in those of bagged fruits, whereas no carotenoids were detected in the flesh (**Figure 1B**; **Supplementary Table S2**). These findings underscore the importance of light in promoting carotenoid biosynthesis in pear peels and reveal the tissue-specific regulation of carotenoid accumulation.

### Transcriptomic and metabolite analyses highlight the role of key genes in β-carotene biosynthesis

Transcriptomic analysis revealed that the expression levels of carotenoid biosynthesis genes, such as *PSY*, *PDS*, and *LCYb*, were greater in the peels of non-bagged fruits than in those of bagged fruits (**Figures 2B, C**). Consistent with these transcriptional differences, HPLC analysis revealed that β-carotene levels were significantly elevated in the non-bagged fruit peel, whereas the other three carotenoids (violaxanthin, 9-cis-violaxanthin and lutein) presented minimal variation between treatments (**Figure 3**).

Previous studies have identified *PSY* as a key regulator of β-carotene biosynthesis, significantly influencing its accumulation in mango fruits (Ma et al., 2021). Consequently, *PSY* was selected as a key candidate gene for functional validation. Transient overexpression and silencing experiments confirmed the role of *PSY* in regulating β-carotene biosynthesis in pear peels (**Figures 4D**, **5C**). These results collectively demonstrate that light exposure enhances *PSY* expression, leading to increased β-carotene accumulation in pear peels.

### AGL8 and LFY proteins cooperatively regulate *PSY* transcription

The *PSY* gene serves as a critical rate-limiting step in the carotenoid biosynthesis pathway, acting as a regulatory switch. Its transcriptional regulation involves both direct and indirect mechanisms. For example, in tomato, transcription factors such as fruitfull1 (FUL1), FUL2, b-box domain protein20 (BBX20), and apetala2a (AP2a) positively regulate *PSY* expression (Fujisawa et al., 2014; Stanley and Yuan, 2019), increasing carotenoid biosynthesis, whereas MADS1 and FYFL act as repressors (Dong et al., 2013).

In this study, transcriptomic analysis revealed that *AGL8*, a MADS-box transcription factor, was highly expressed in the non-bagged fruit peels (**Figure 6A**). LCI and dual-luciferase assays confirmed that AGL8 can directly bind to the *PSY* promoter and activate its transcription (**Figures 6C, D**). However, yeast-based transcriptional activation assays indicated that AGL8 alone does not exhibit strong activation activity (**Supplementary Figure S7**), suggesting the involvement of an additional coregulator.

Protein interaction screening identified LFY, a transcription factor associated with flowering, as an interaction partner of AGL8 (Hu et al., 2023). Dual-luciferase and LCI assays demonstrated that AGL8 interacts with LFY and that their cooperative action significantly enhances *PSY* transcriptional activation (**Figures 6**, **7**). This cooperative regulatory mechanism represents a novel pathway through which AGL8 and LFY jointly modulate carotenoid biosynthesis by activating *PSY*.

## Conclusion

The bagging treatment significantly reduced the content of carotenoids, particularly β-carotene, in the peel. Transcriptomic analysis revealed a notable difference in *PSY* expression between bagging treatments, and transient transformation experiments confirmed that *PSY* plays a key role in regulating β-carotene accumulation in pear peels. Furthermore, interaction studies revealed that AGL8 interacts with the LFY protein to coactivate the transcription of *PSY*. These findings offer valuable insights into the molecular mechanisms underlying the light-mediated regulation of fruit pigmentation, with potential applications in improving pear fruit quality through light management and molecular breeding strategies.

## Data Availability

The datasets presented in this study can be found in online repositories. The RNA-seq data were submitted to the National Center for Biotechnology Information (NCBI) Short Read Archive (SRA) Sequence Database. The raw data can be accessed via the accession number PRJNA928225 in NCBI.

## Supplementary material

Supplementary Data Sheet S1 The quality score (Q30) and GC content of all the samples.

Supplementary Data Sheet S2 A total of 14,643 DEGs were identified between bagged and non-bagged fruits peel in ‘Cuiguan’ pear.
