# Supplementary material for: Light-mediated activation of PpPSY enhances β-carotene accumulation in pear fruit peel
Source: Front Plant Sci. 2025 Feb 28;16:1542830. doi: 10.3389/fpls.2025.1542830 (PMC11906342; doi:10.3389/fpls.2025.1542830)
Supplement: Supplementary file 3 [file Image1.pdf]

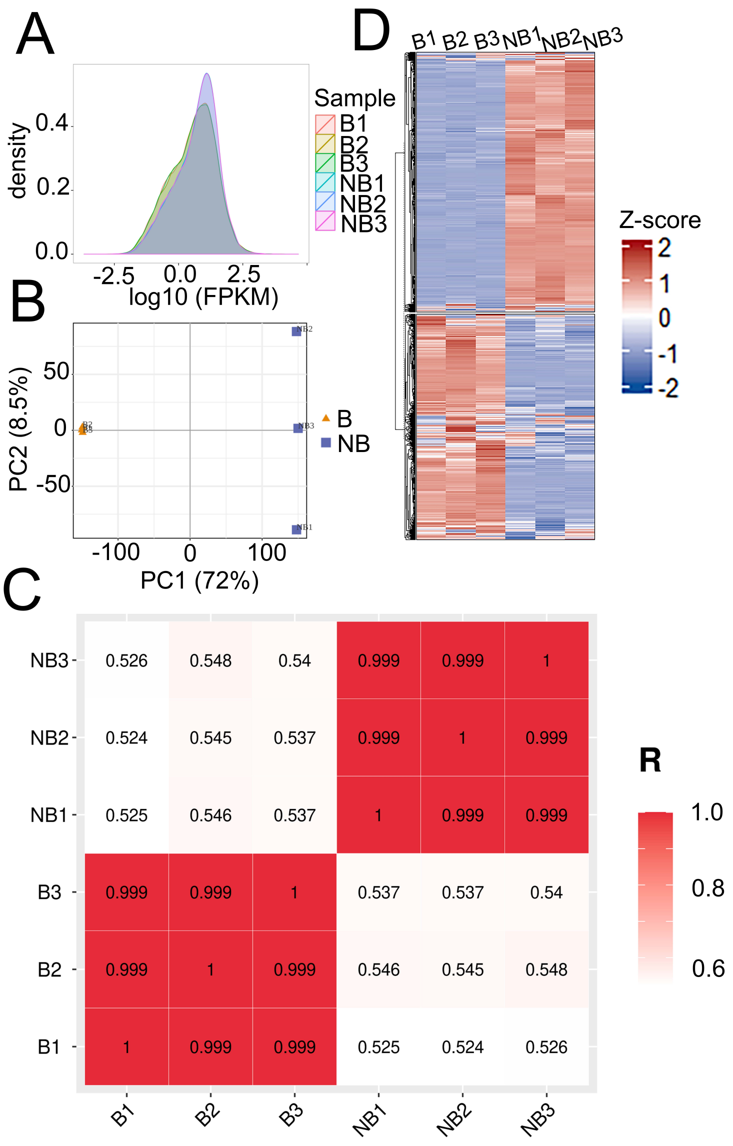

	B1	B2	B3	NB1	NB2	NB3
NB3	0.526	0.548	0.54	0.999	0.999	1
NB2	0.524	0.545	0.537	0.999	1	0.999
NB1	0.525	0.546	0.537	1	0.999	0.999
B3	0.999	0.999	1	0.537	0.537	0.54
B2	0.999	1	0.999	0.546	0.545	0.548
B1	1	0.999	0.999	0.525	0.524	0.526

Supplemental Figure S1. Overall analysis of transcriptome data. (A) Comparison chart of FPKM density distribution for each sample. The curves of different colors represent different samples. The x-axis of the points on the curves indicates the logarithmic value of the FPKM for the corresponding samples, while the y-axis represents the probability density. (B) Principal component analysis (PCA) plot. Each point represents an independent biological replicate. (C) Expression correlation heatmap. In the heatmap, each colored block represents the correlation value between the two samples indicated on the x and y axes. Higher values indicate stronger correlations. (D) Differential gene heatmap.

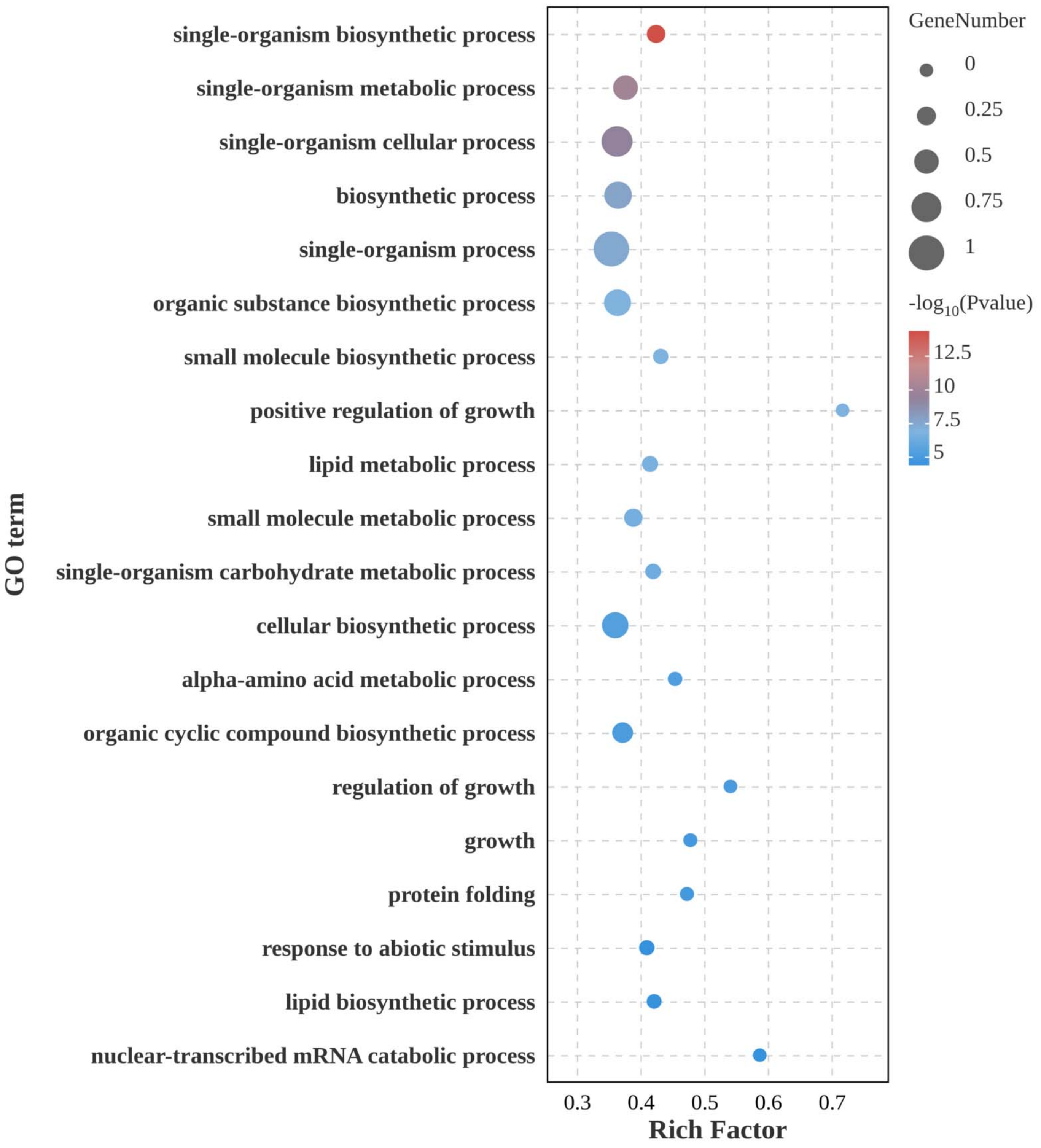

Supplemental Figure S2. GO enrichment analysis for the differential genes in NB and B fruit peel in pear. The circle size indicted the Differential genes count, and the circle color indicated q value.

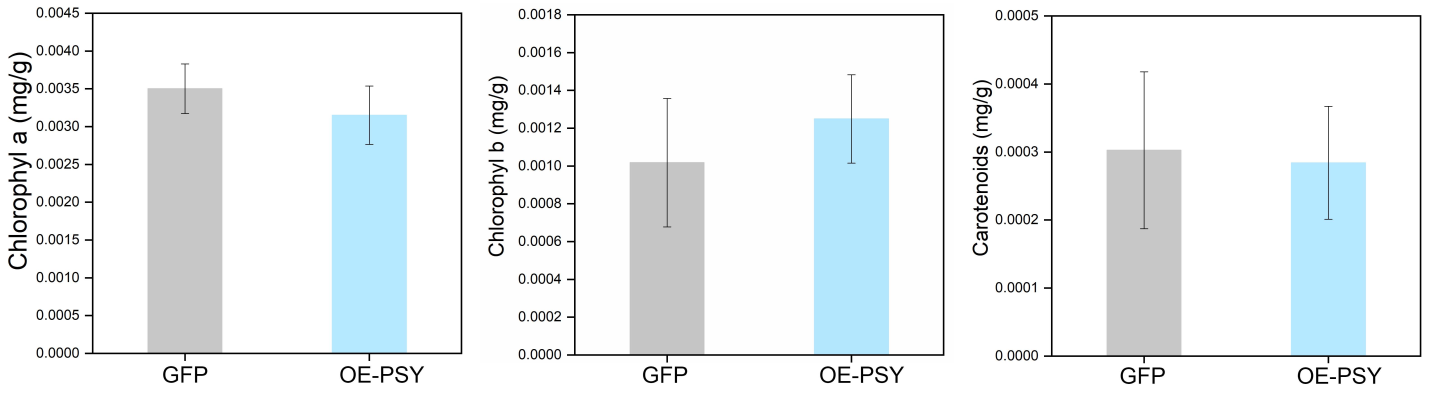

Supplemental Figure S3. Measurement of carotenoids and chlorophyll content in the 0 day after injection (DAI) for the overexpression of *PSY* in pear.

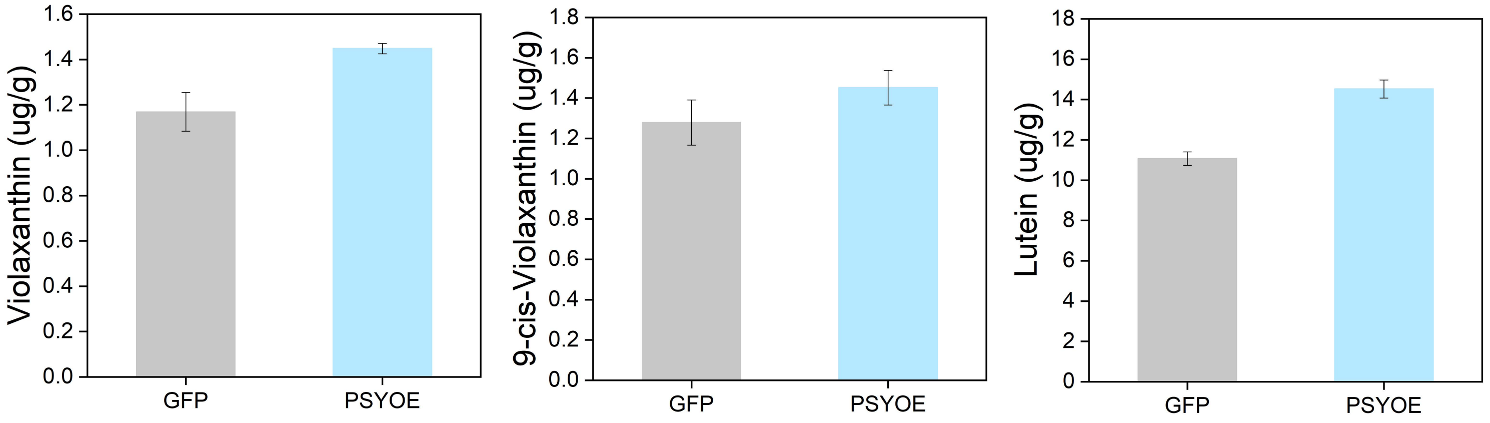

Supplemental Figure S4. HPLC analysis of GFP and PSYOE fruit peel.

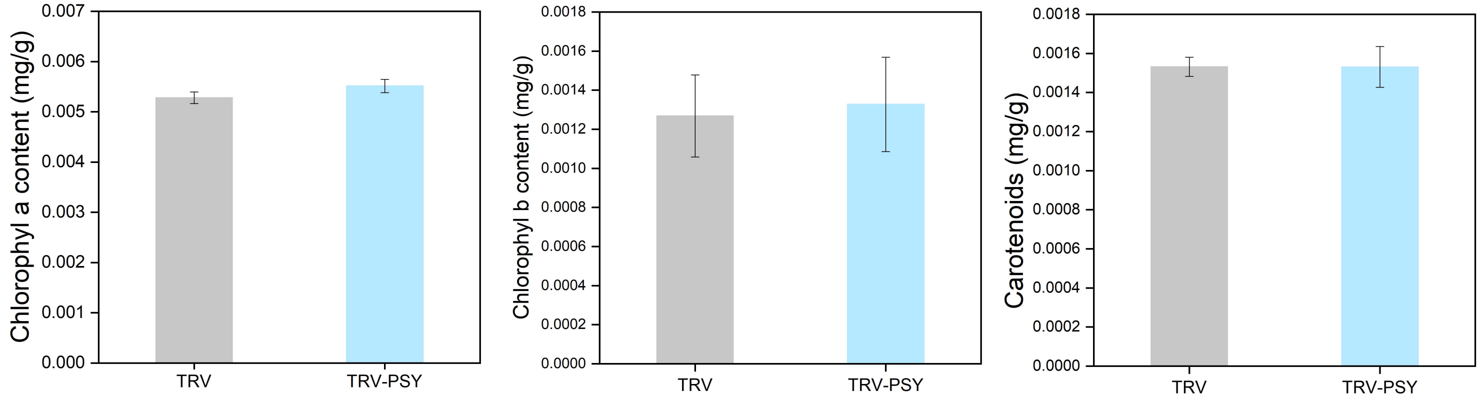

Supplemental Figure S5. Measurement of carotenoids and chlorophyll content in the DAI 0 for the silencing of PSY in pear.

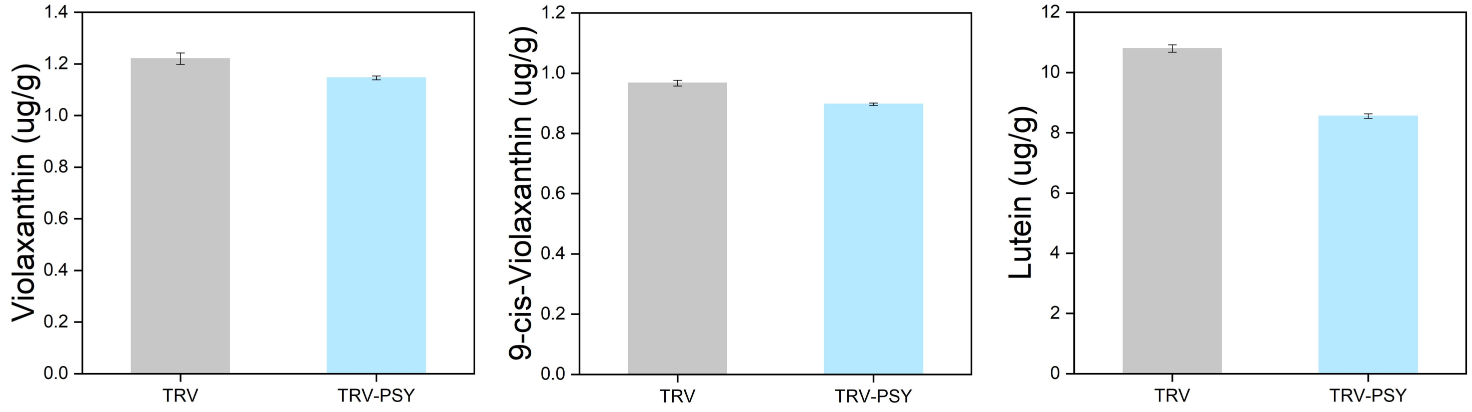

Supplemental Figure S6. HPLC analysis of TRV and TRV-PSY fruit peel.

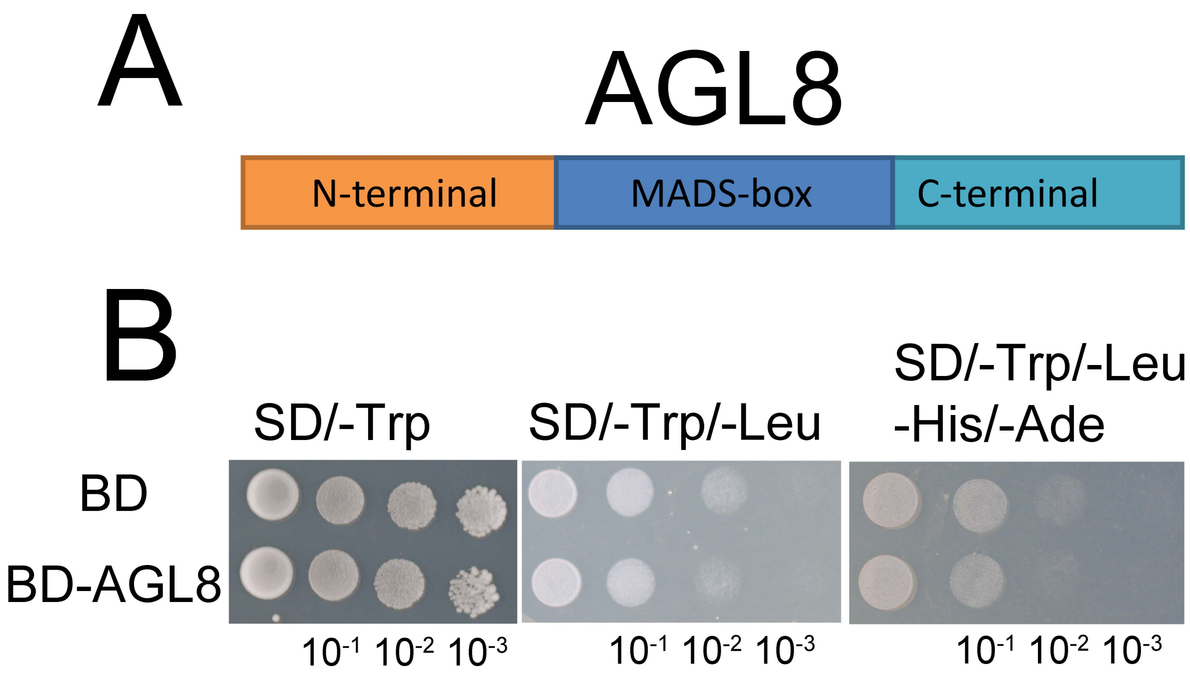

Supplemental Figure S7. Transcriptional activation of AGL8 in the AH109 yeast cell.

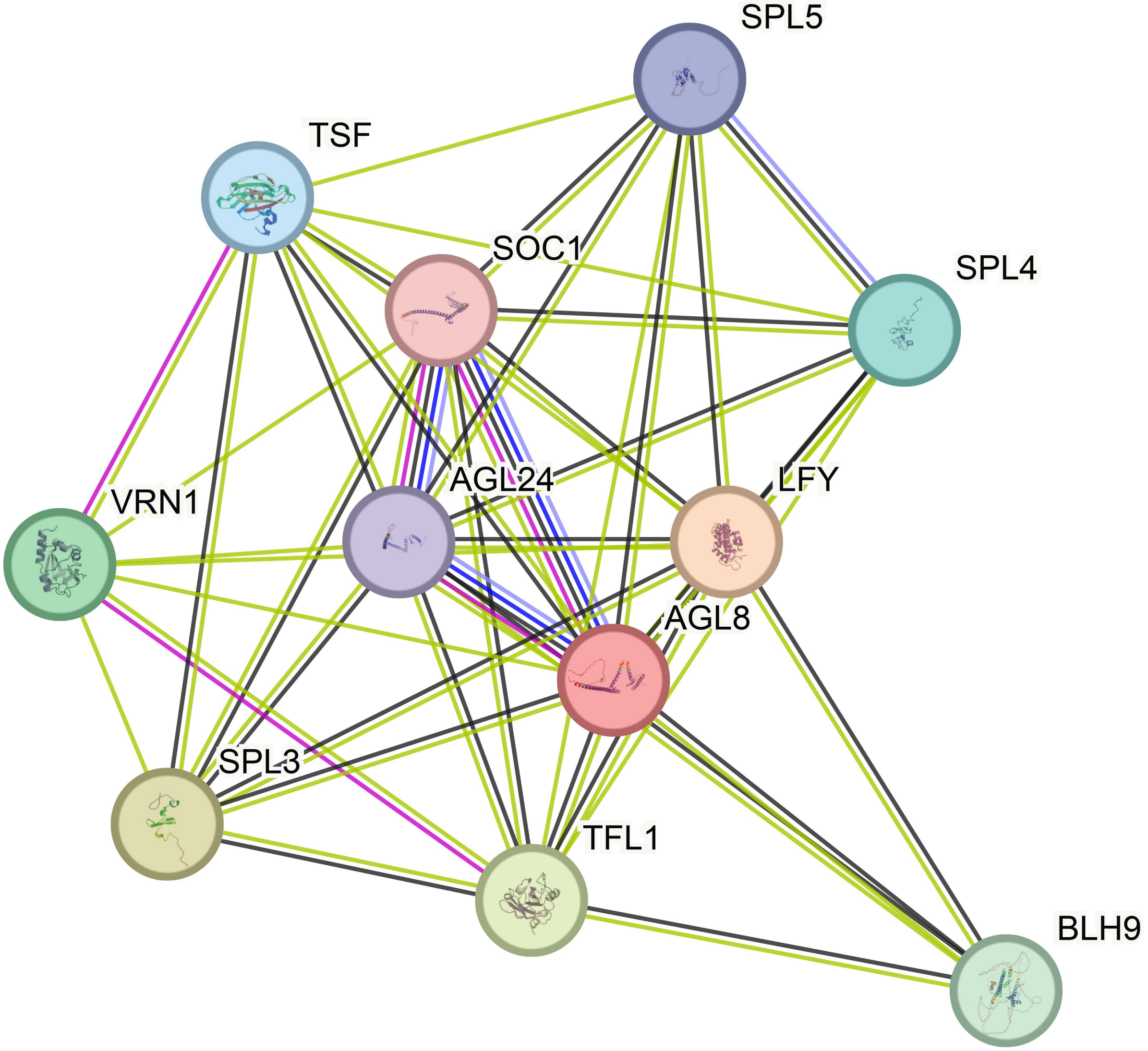

Supplemental Figure S8. Candidate proteins for AGL8 interaction. Nodes: network nodes represent proteins; Edges: edges represent protein-protein associations; Color lines represent that there may be interaction between proteins.
